# Tools of the trade: studying actin in zebrafish

**DOI:** 10.1007/s00418-020-01932-3

**Published:** 2020-10-23

**Authors:** Clyde Savio Pinto, Masanori Mishima, Karuna Sampath

**Affiliations:** 1 Division of Biomedical Sciences, Warwick Medical School, University of Warwick, Coventry, CV4 7AL UK; 2 Centre for Mechanochemical Cell Biology, University of Warwick, Coventry, CV4 7AL UK; 3 Center for Early Life, Warwick Medical School, University of Warwick, Coventry, CV4 7AL UK

**Keywords:** Zebrafish, Actin, Cytoskeleton, Imaging, Inhibitors

## Abstract

Actin is a conserved cytoskeletal protein with essential functions. Here, we review the state-of-the-art reagents, tools and methods used to probe actin biology and functions in zebrafish embryo and larvae. We also discuss specific cell types and tissues where the study of actin in zebrafish has provided new insights into its functions.

## Introduction

Actin is a cytoskeletal protein that is highly abundant inside animal cells. It is an asymmetric molecule including the site of the nucleotide-binding cleft and polymerises to form a double helical filament (dos Remedios et al. 2003). Polymerisation occurs in a polarised fashion, with a higher rate of monomer addition at the barbed end than the pointed end and generates flexible filaments (Pollard 1986). Filaments also age with change of nucleotide state from ATP to ADP (Pollard et al. 2000). The filaments are organised into higher order structures and can generate force, provide stiffness and serve as tracks for the movement of cargo.

There are three classes of actin—the alpha, beta and gamma actin—in mammals. The alpha actins are found in muscle cells and are of three types: skeletal, cardiac and smooth. All three types of muscle actins are present in zebrafish, with approximately 99% identity to the corresponding human proteins. The other mammalian actins are found predominantly in non-muscle cells and are called cytoplasmic actins (Rubenstein 1990).

**Fig. 1:**
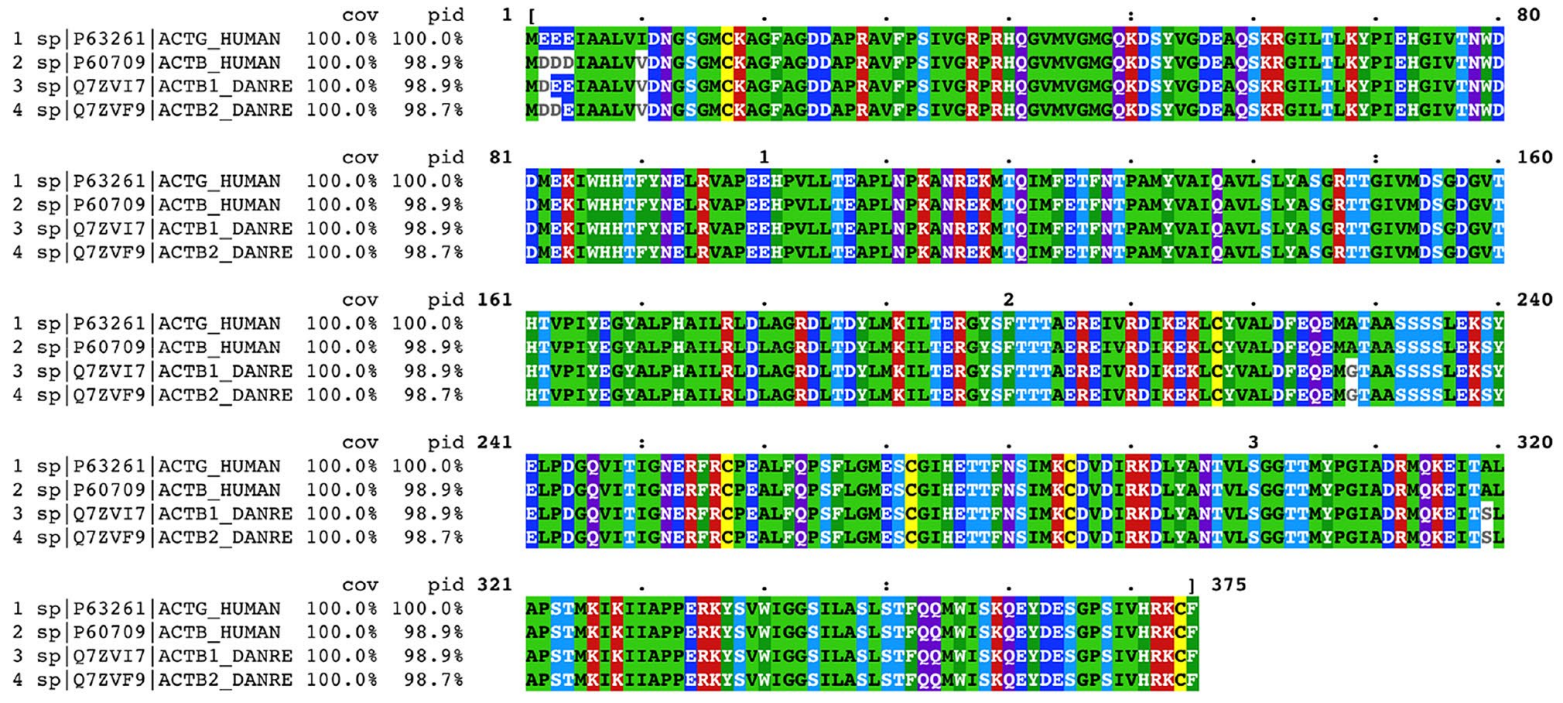
Clustal omega based comparison of the human and zebrafish (Danio rerio) cytoplasmic actins. The sequences were obtained from uniprot (UniProt 2019), aligned using clustal omega (Sievers et al. 2011), and viewed using Mview (Brown et al. 1998). cov is the percent coverage and pid is the percent identity relative to the reference sequence which is ACTG_HUMAN

In zebrafish, there are two cytoplasmic actins, namely actin B1 (*actb1*) and actin B2 (*actb2*), based upon a uniprot analysis (UniProt 2019). They differ by only one amino acid at the position 3, with glutamic acid in B1 and aspartic acid residue in B2 (Fig. 1). B1 and B2 share significant homology with human actins beta and gamma, with only 3–5 amino acid changes. These include differences in the variable N-terminus region of actin which also distinguishes Beta from Gamma human actin. How these differences modify the properties of actin has not yet been studied, but considering the sequence similarity of the zebrafish actins to both Beta and Gamma actins, it is unclear if the zebrafish cytoplasmic actins can be classified under either of these two classes or whether they represent evolutionary intermediates.

Post-translational modifications can modify the repertoire of a protein’s functions (Wang et al. 2014). Actin is also post-translationally modified resulting in alterations of its properties (Varland et al. 2019). In mammalian systems, the Beta isoform of actin is post-translationally arginylated in vivo, however, this modification does not occur on the gamma isoform (Kashina 2006). Also, mammalian actin is acetylated (Varland et al. 2019). Whether zebrafish actins are post-translationally modified, and what types of modifications on actin are present is currently unknown. Additionally, the molecular properties of zebrafish actins have not been studied.

Recently, Scheid et al. (2017) purified zebrafish skeletal muscle actins and demonstrated their filament forming and myosin interacting capacities in vitro. Despite a lack of data regarding the biochemical properties of zebrafish cytoplasmic actins, considering the fact that they share almost complete sequence homology with the well-studied mammalian actins, it is likely that their properties and structure are similar to the mammalian proteins. Since actins are an essential part of the cytoskeleton that generate physical forces and mediate cell shape changes, they have been imaged in zebrafish embryos and larvae, and actins properties have been modified in many ways.

Zebrafish are widely known to possess several features that are advantageous for the study of various aspects of biology especially cell and developmental biology as well as disease pathology. Some of these advantages are that they have a high fecundity, transparent embryos, rapid development, susceptibility to genetic, chemical and physical manipulations and offer relatively easy high-resolution imaging in a live vertebrate organism (Vacaru et al. 2014; Torraca and Mostowy 2018; Meyers 2018). These significant benefits make zebrafish attractive for the study of actin and its regulation in various cells and tissues, in an in vivo vertebrate setting. In this review, we provide an overview of the tools and techniques that can be used to visualise and alter zebrafish actins.

## The visualisation of actin in cells

### Phalloidin staining

Phalloidin originally isolated from *Amanita phalloides* (death cap mushroom) has a high affinity for F-actin. It is a bicyclic peptide and is not membrane permeable (Wulf et al. 1979). It is used either in live or fixed cells. In live cells or in vitro, phalloidin can be used to stabilize actin filaments by preventing depolymerisation (Melak et al. 2017; Coluccio and Tilney 1984). This stabilisation can be used either to study the effects of stabilizing actin in a given context or to preserve actin in techniques in which actin is otherwise prone to disruption such as electron microscopy. However, as phalloidin does not penetrate membranes, it needs to be delivered into cells e.g. by microinjection (Planques et al. 1991), or used as a membrane permeable form called phalloidin-oleate (Dutta and Kumar Sinha 2015).

In electron microscopy, fixation approaches popularised by Svitkina (2016) cells in which actin is to be preserved are first unroofed to remove their membrane using a buffer containing Triton X-100 and phalloidin, sometimes also containing polyethylene glycol (PEG). The phalloidin and PEG are thought to protect and stabilize the actin cytoskeleton during fixation and downstream processing. This is followed by fixation with glutaraldehyde, uranyl acetate and platinum replica plating for TEM or staining by the OTOTO method for SEM (Pinto et al. 2019; Svitkina 2016). Previous studies reported the reliable use of this method for fish cells in culture (Svitkina et al. 1997), and recently, this approach has also been used on 2 dpf zebrafish larvae, followed by SEM to examine the actin microridge in zebrafish (Pinto et al. 2019).

The most widespread use of phalloidin is in the form of fluorescent derivatives used for the staining of F-actin in fixed cells, making it a quintessential part of the actin imaging toolkit. Phalloidin staining is typically performed on paraformaldehyde (PFA) fixed samples without methanol upgradation, as methanol adversely affects the preservation and detection of actin (Melak et al. 2017). It can be used in one of two ways. For cells in culture and for surface cells in zebrafish, the fixative itself is sufficient for permeabilising cells and no detergent is required. Higher concentrations of phalloidin might be required for this technique and normally a dilution of 1:40 (using an approximately 66 µM stock) over 3-4 h is sufficient for good labelling of zebrafish peridermal actin (Pinto et al. 2019).

For staining the epidermis as well as deeper tissue, however, detergent-based permeabilization is routinely employed normally using Triton X-100 and phalloidin dilutions from 1:40 to 1:400 as appropriate (Pinto et al. 2019). Staining times need to be modified to ensure that the tissue of interest is stained. For older animals or internal tissues, it is also possible to either dissect the tissue of interest or section the sample by means of a vibratome, microtome or cryostat (Sidhaye et al. 2016). It should be noted, however, that both fixation as well as the expression of live imaging probes have the potential to generate artefacts. Expression of high levels of molecules such as LifeAct in cells can stabilize actin filaments (Flores et al. 2019). On the other hand, while 4% PFA in PBS is routinely used for a number of staining methods, it might be inadequate for actin preservation under some conditions and more optimal fixation methods employing buffers that contain, for instance, EGTA and magnesium (e.g. PEM) may be required (Pereira et al. 2019; Wheatley and Wang 1998; Leyton-Puig et al. 2016; Heuser and Kirschner 1980). These factors should be kept in mind and optimised during experimentation and analysis to obtain reliable results.

### LifeAct and Utrophin-CH domain

LifeAct and Utrophin-CH domain have been the most widely used probes for the live imaging of actin in zebrafish. LifeAct is a small 17 amino acid actin-binding peptide from the Abp140 protein of *Saccharomyces cerevisiae* with the amino acid sequence ‘MGVADLIKKFESISKEE’ (Riedl et al. 2008). The small size facilitates cloning as a codon optimised LifeAct sequence can be included directly on a long primer for fusion to a fluorescent protein or tag of interest. It has been shown to reliably bind to F-actin in various contexts and so is the go to probe for imaging actin dynamics in vivo, however, it might also bind G-actin (Melak et al. 2017). Care should be exercised when using LifeAct as at high concentrations it has been demonstrated to alter actin dynamics in vivo as well as can have an overall impact on the cytoskeleton and biology of the cell (Flores et al. 2019).

The calponin homology domain of human Utrophin (Burkel et al. 2007) is also widely used to label F-actin. This domain does not bind to G-actin, and in this sense, is considered superior to LifeAct for the purpose of imaging only F-actin (Winder et al. 1995). It is relatively large in size including the first 261 amino acids of human UTROPHIN. Zebrafish lines for the utrophinCH domain exist and are widely used (Behrndt et al. 2012).

### SiR-actin

This actin probe consists of silicon-rhodamine (SiR) conjugated desbromo-desmethyl-jasplakinolide (Lukinavicius et al. 2014). The key advantage of SiR-Actin is that it is cell permeable and photostable (Lukinavicius et al. 2014) and, therefore, in the context of the external surface of zebrafish, SiR-Actin facilitates live imaging of endogenous actin. For instance, it has been used to observe actin in the retina of zebrafish (Matejcic et al. 2018). SiR and SiR-Actin emit fluorescence in the far-red wavelength (Lukinavicius et al. 2013, 2014). Its photostability make it a useful tool to probe Actin through super resolution microscopy techniques such as STED (Lukinavicius et al. 2014).

### Actin chromobody

Nanobodies from Camelidae can be genetically encoded with fluorescent fusion tags and expressed in cells. Such ‘chromobodies’ against actin have been generated and can serve as powerful tools to image and study actin structures in vivo (Panza et al. 2015). Actin chromobodies have been used in zebrafish and stable lines have been generated. This can serve as an excellent genetically encoded tool to study live actin dynamics in vivo and can mark both F and G actin (Panza et al. 2015). However, this tool has not yet been widely exploited, and can potentially provide new insights into actin dynamics in zebrafish.

### Injection of labelled actins

One of the significant strengths of zebrafish, especially when studying early development is the ability to inject protein/RNA/DNA into the 1 cell stage embryo or in one or more cells at the 16 cells stage embryo. At these stages, the cells are sufficiently large, allowing microinjection with relative ease under a stereo microscope (Gupta and Sonawane 2020; Rosen et al. 2009). It is also possible to inject the yolk cell at later time points to image and manipulate components and study the contribution of this cell to developmental processes.

Injecting protein or RNA or DNA each have their advantages or disadvantages. Protein injections allow the study of these molecules from the timepoint of injection till their degradation. For instance, the injection of LifeAct-RFP protein will allow the immediate detection of F-actin in vivo. RNA encoding such actin imaging or manipulating molecules, however, will only express post maternal zygotic transition, but this should allow the imaging of these molecules for a longer time duration till mRNA and protein are degraded. mRNAs with special UTR’s can also allow tissue-specific expression such as with the nanos 3′UTR that is not degraded in the germ line and so allows the imaging or manipulation of PGC’s. Finally, plasmid DNA can also be used for transient transgenesis, either with or without transposable elements. However, plasmid DNA expression is typically mosaic, patchy, and expression is clonal, which might be advantageous in some contexts, but may not be useful if widespread expression is desired. The use of transposable elements and transposase results in the integration of the sequence of interest into the zebrafish genome and long-term expression (Kawakami 2007). Plasmids can be generated with promoters of interest such as the Actb promoter or the CMV promoter, that express robustly in multiple cell types.

While bulky tags such as GFP are known to alter actin’s polymerisation properties especially when fused to the N-terminus of actin (Nagasaki et al. 2017), rhodamine or other small fluorescent dyes can be used to label actin decreasing the negative effects of bulky groups and allowing the visualisation of actin in vivo. Typically, human platelet actin or actin from muscle sources of various animals such as rabbits is used for these purposes. One of the biggest advantages of labelled actin is that it allows the direct assay of actin dynamics either by means of speckle microscopy or by other methods such as FRAP (Waterman-Storer et al. 1998). The labelled actins can be introduced into zebrafish embryos via microinjection (Cheng et al. 2004). Recently, a robust new method for the expression of homogenous functional actins from recombinant yeast has been developed (Hatano et al. 2018, 2020). Actin expressed, purified and labelled in this way has been tested in zebrafish embryos (Hatano et al. 2018). However, as yet mammalian but not zebrafish actins have been used in such studies.

### C-terminus of Moesin

The C-terminus of the actin-binding protein, Moesin, has been used in zebrafish to image actin in vivo, through a transgenic line that allows the visualisation of actin filaments and actin dynamics during oogenesis and early development (Nukada et al. 2015).

## Modulation of the actin cytoskeleton by means of inhibitors

### Cytochalasin

Cytochalasins act by capping the barbed end of actin and preventing the addition as well as the disassociation of actin monomers at this end (Brown and Spudich 1981; MacLean-Fletcher and Pollard 1980). A wide range of cytochalasins are available, which are soluble in DMSO and can be diluted into the E3, Danieau buffers or fish water prior to use with fish embryos. Recently, a photoactivable or caged form of cytochalasin D that allows the spatio-temporal control of cytochalasin targeting has also been described (Latorre et al. 2018).

### Latrunculins

Latrunculins are small molecule inhibitors that affect the actin cytoskeleton in multiple ways including the sequestration of monomers and increasing the rate of filament breakdown (Fujiwara et al. 2018). They have been very widely used in zebrafish. Latrunculins are soluble in DMSO and can be stored as 1 mM stocks at − 20 °C, and can be diluted before use. A 2 μM working solution of latrunculin A for 20 min treatment is a good starting point (Raman et al. 2016). It should be noted that all treatments need to be titrated and optimised to facilitate the investigation of the processes under study as different treatment regimens might be toxic or lethal to the animal or to particular cell types.

### Blebbistatin

Myosin 2 activity being the primary driver of acto-myosin contractility, inhibition of its activity is widely employed in zebrafish biology. Blebbistatin is an inhibitor that binds to myosin in the ADP + Pi state and reduces the rate of release of Pi, in this state myosin has a low affinity for actin (Kovacs et al. 2004). Blebbistatin is inactivated by light of wavelengths below 488 nm, which can be used to reactivate myosin activity, but should also be considered when live imaging GFP and similar fluorophores in the presence of blebbistatin (Sakamoto et al. 2005). Blebbistatin is soluble in DMSO and can be stored as a 100 mM stock solution at − 20 °C. A suitable starting concentration is 10 μM, for a duration of 1.5 h (Raman et al. 2016).

### Arp2/3 complex inhibitors

One of the key regulators of actin structure is the Arp2/3 complex. It is a seven subunit complex that serves as a nucleator of new actin filaments (Pollard et al. 2000). It is the key nucleator of branched actin filaments that are found in actin structures such as lamellipodia, growth cones as well as endocytic actin patches (Korobova and Svitkina 2008; Wu et al. 2012; Galletta et al. 2008). Specific inhibitors of the Arp2/3 complex were published by the Pollard group in 2009 (Nolen et al. 2009). One of the most commonly used Arp2/3 complex inhibitors is CK-666 which works by stabilizing the inactive state of the complex. Another, drug CK-869 works by destabilizing the active state of the Arp2–Arp3 dimer (Hetrick et al. 2013). These two molecules are soluble in DMSO and can be stored at − 20 °C. They are often used with their inactive drug controls, CK-689 for CK-666 and CK-312 for CK-869. A good starting concentration to try is 100 μM for 1 h (Pinto et al. 2019).

### SMIFH2

Another critical nucleator of actin is the group of proteins known as formins. They not only nucleate actin but also elongate actin filaments and have wide importance in multiple actin structures that might be made of branched or unbranched filaments (Breitsprecher and Goode 2013). While there are 15 formin proteins in mammals a common structural element in formins is the formin homology 2 domain (Schonichen and Geyer 2010) [An Interpro taxonomy search for formin, FH2 domain superfamily shows 55 proteins in *Danio rerio* and 71 in *Homo sapiens* (*Mitchell et al.* 2019)]. It is this domain that is targeted by the cell permeable small molecule inhibitor SMIFH2 (Rizvi et al. 2009). SMIFH2 is soluble in DMSO and in our hands works best when prepared fresh from powder immediately prior to usage. Due to the large number of formins, it might not always be possible to ascertain which formins are active in your tissue of interest a priori. In this context, it might be worthwhile testing the effect of SMIFH2 to determine if formins play a role in the process of interest, followed by more intensive identification and manipulation of the specific formins involved. However, it should be noted that concentrations greater than 5 μM for long time periods such as 4 h have strong toxic effects on various organ systems in zebrafish larvae (LeCorgne et al. 2018).

## Lipids

The membrane is known to exert a profound effect on the actin cytoskeleton (Saarikangas et al. 2010). These effects are either on account of the effects of the membrane on actin-binding proteins or by directly affecting actin filaments by changing properties of the actin membrane interaction (Vasanji et al. 2004; Yin and Janmey 2003). This review will briefly touch upon 3 lipids, namely phosphotidylinositol (PI)-4,5-biphosphate (PIP2), phosphotidylinositol-3,4,5-triphosphate (PIP3) and cholesterol.

Cholesterol regulates the physical properties of the membrane, with higher levels leading to less fluid membranes with altered stiffness (Cooper 1978; Ayee and Levitan 2016). It has been shown that the ability of actin to deform membranes requires intermediate levels of cholesterol with levels too high or too low reducing the ability of actin to deform the membrane (Vasanji et al. 2004). Cholesterol is also enriched to appropriate levels in locations that are actin rich such as the leading edge of migrating cells that have lamellipodia or the apical surface of MDCK cells that have microvilli (Vasanji et al. 2004; Gerl et al. 2012).

Filipin is used as a common dye for the imaging of cholesterol in cells. It is sensitive to bleaching and so should be imaged as soon as possible once staining is complete (Boutte et al. 2011). The levels of cholesterols can be decreased by incubating larvae with methyl-β-cyclodextrin. Methyl-β-cyclodextrin is water soluble which makes its use in fish media straightforward. It is preferred to make up the solution freshly before use with a good starting working concentration being 2.5 mM for 30 min, concentrations of 10 mM for acute treatments and 0.5 mM for long-term treatments have also been used (Maerz et al. 2019). Methyl-β-cyclodextrin can also be saturated with cholesterol to make it a cholesterol donor that will increase the levels of cholesterol in cells (Zidovetzki and Levitan 2007).

PIP2 regulates several aspects of actin polymerisation and actin-binding protein activity. PIP2 plays a role in activating WASP proteins that act as nucleation promoting factors for the Arp2/3 complex as well as Ezrin that act as actin membrane linkers (Higgs and Pollard 2000; Jayasundar et al. 2012). PIP2 can also inhibit the F-actin severing activity of gelsolin (Janmey and Stossel 1987). Apart from these, it can regulate a whole host of actin-binding proteins (Yin and Janmey 2003). PIP2 can be imaged by means of a sensor PLCδ1-PH which is the PH domain of phospholipase Cδ1 domain, fused to a fluorescent protein (Yin and Janmey 2003; Watt et al. 2002). This sensor can be genetically encoded and has been used in zebrafish (Gong et al. 2017). It should be noted that this sensor has a preference for PIP2 and will be enriched in PIP2 rich domains, but not normally completely absent from other parts of the plasma membrane.

PIP3 can regulate the RhoGTPases such as Rac that stimulates the polymerisation of actin (Innocenti et al. 2003). PIP3 is synthesized by PI3Kinase and the PH domain of the Akt protein binds to it (Yin and Janmey 2003). Akt-PH-GFP can thus be used as a sensor of PIP3 levels in the same way as PLCδ1-PH for PIP2 and has been used in zebrafish (Yoo et al. 2010). Both PIP2 and PIP3 can be added exogenously to cells (Strawbridge and Elmendorf 2005; Martin-Belmonte et al. 2007; Gassama-Diagne et al. 2006), though this method might be difficult when studying internal tissues in zebrafish, for such cases, it might be possible to genetically alter the levels and/or activity of PIP2 or PIP3 synthesizing enzymes.

## Genetic tools to study actin biology

### Rho GTPase

Rho GTPases are a family of small GTPases that have wide ranging effects on the actin cytoskeleton and are important regulators of the actin cytoskeleton. Historically, by means of classic experiments from the Hall lab, we know that the three major classes of Rho GTPases have divergent roles (Ridley and Hall 1992; Ridley et al. 1992; Nobes and Hall 1995). Rho GTPases undergo a GTPase cycle with the GTP bound form being active, and the GDP bound form being inactive (Etienne-Manneville and Hall 2002). The Rho GTPases in the active state can interact with a wide variety of effectors for instance Rho GTPase interacts with mDiaphanous (Schwartz 2004). Due to this, the localisation of active Rho GTPase forms inside cells is of interest.

Fluorescence/Förster resonance energy transfer (FRET)-based sensor tools that identify the active state of Rho GTPases have been developed and reveal the localisation of active Rho GTPases (Hodgson et al. 2010). These have been used in zebrafish, in various contexts such as their role in the migration of primordial germ cells in developing zebrafish (Kardash et al. 2010).

Apart from observing the in vivo activity of Rho GTPases in cells, manipulating their activity is also possible by means of dominant negative and constitutively active forms of the Rho GTPases (Kardash et al. 2010). Photoactivable forms of the Rac GTPase have also been used providing fine spatio-temporal control over Rac activity (Yoo et al. 2010).

### Myosin 2

Non-muscle myosin IIs (NMMII) have several roles inside the cell with respect to actin. They are involved in actin contraction, crosslinking as well as disassembly of actin (Sonal et al. 2018; Laevsky and Knecht 2003). These acto-myosin interactions play a major role in generating cellular and tissue level forces (Heisenberg and Bellaiche 2013). Consequently, manipulation of the acto-myosin interaction is an important tool in the hands of cell biologist that study the actin cytoskeleton. This manipulation can be by means of drugs such as blebbistatin as well as genetic tools.

NMMIIs are known to be regulated by phosphorylation of their regulatory light chain (Watanabe et al. 2007), with phyosphorylation by the myosin light chain kinase or Rho-kinase leading to activation (Heissler and Manstein 2013). Constitutively active forms of the myosin light chain kinase have been used widely to activate myosin and increase acto-myosin contractility inside cells (Blaser et al. 2006). NMMII can also be regulated indirectly by regulating Rho GTPase and Rho-kinase. The phosphorylation state of NMMII can also be monitored in cells using FRET based sensors similar to those used in *C. elegans* (Markwardt et al. 2018).

### Arp2/3 complex

The Arp2/3 complex is a weak nucleator of actin (Goode et al. 2001). Its nucleation activity is greatly enhanced on activation by nucleation promoting factors such as WASP (Rohatgi et al. 1999). The VCA domain of WASP can activate the Arp2/3 complex, and can be used as a tool to increase its activity in vivo (Rohatgi et al. 1999). On the other hand, the protein Arpin is an inhibitor of the Arp2/3 complex and has been used as a tool to decrease its activity in zebrafish (Dang et al. 2013).

### Actin depolymerising agents

Recently, novel genetically encoded tools that lead to the breakdown of actin have been generated. They have been christened ‘DeActs’ and consist of parts of proteins such as gelsolin coupled to GFP for visualisation. They have been used and shown to be functional in vivo in mouse and *C. elegans* (Harterink et al. 2017). The F actin network can also be remodelled by genetically modified cofilins that are fused to light activated domains. Such approaches would further enhance the genetic toolkit of actin regulators in zebrafish (Hughes and Lawrence 2014; Stone et al. 2019).

### Mutants

Mutants for a few actin-binding proteins and actins such as Cofilin, capping protein β-subunit, L-plastin, WASP and skeletal α-actin exist and several morpholinos such as that against zDia2 have been used in the past (Kell et al. 2018; Amsterdam et al. 2004; Cvejic et al. 2008; Sztal et al. 2018; Lai et al. 2008). It should be noted that with advances in genome editing as well as transgenesis, it is now possible to make mutants as well as transgenic lines of almost any gene of interest in zebrafish.

## Actin model systems

The rapid development of zebrafish organ systems within a few days of development allows the study of actin in specific contexts and can provide insight into different aspects of actin regulation in specific cell types and tissue. These include the study of migration of neurons, collective migration such as that by the lateral line primordia as well as single cell migration of germ or immune cells, and the development of various muscles (Deng and Huttenlocher 2012; Olson and Nechiporuk 2018; Paksa and Raz 2015; Rocha et al. 2020; Sanger et al. 2009). Here, we discuss examples of a few selected model cell types and tissues in zebrafish, in which actin regulation can be studied in-depth.

### Microridges

Nowhere is the benefit of studying actin in zebrafish more apparent than in the case of the actin microridge. The actin microridge is a widely distributed, labyrinthine actin based apical membrane protrusion (Depasquale 2018). It is found on non-keratinising stratified epithelia such as that of the oral or vaginal mucosa (Eroschenko and Osman 1986; Uehara et al. 1991; Saito and Itoh 1993; Depasquale 2018). Likely owing to its internal nature and non-obvious or non-critical function in mammals, it has not been widely studied. However, in the fish and frog systems, the microridge is an epidermal structure and is present on the outermost surface of the animal apart from various internal tissues (Hawkes 1974; Schliwa 1975; Bereiter-Hahn et al. 1979). While most studies on fish from the 1970s to the 1990s focussed on carp, guppy or seahorse, more recently with the advent of zebrafish as a model system, there has been an increased interest in the zebrafish model from a microridge context (Hawkes 1974; Schliwa 1975; Bereiter-Hahn et al. 1979; Uehara et al. 1991; Sharma et al. 2005; Lam et al. 2015; Raman et al. 2016; Pinto et al. 2019; van Loon et al. 2020; Inaba et al. 2020).

The actin microridge in zebrafish offers the benefits of genetic tractability, ease of imaging as well as the easy access of the surface structure to chemical or physical perturbations (Lam et al. 2015; Raman et al. 2016). Consequently, the zebrafish system has provided considerable novel insight to the microridge structure and function. It has been proven using the inhibitors of the Arp2/3 complex as well as immunolocalisation that the microridge is an Arp2/3 dependent structure (Lam et al. 2015; Pinto et al. 2019). Using electron tomography and SEM of detergent extracted samples, it was shown that the zebrafish peridermal actin microridge is a branched actin structure with an actin and keratin terminal web (Pinto et al. 2019). The mechanisms of pattern generation via cytoskeletal contractility, myosin activity and the balance and interplay between the actin and keratin cytoskeletons in the microridge have also been demonstrated with various live imaging techniques using probes such as LifeAct as well as laser ablation, inhibitor studies as well as CA-MLCK (van Loon et al. 2020; Raman et al. 2016; Inaba et al. 2020). Apart from direct studies of the actin cytoskeleton in microridges, the effect that cell polarity regulators have on the actin cytoskeleton have also been demonstrated using the zebrafish peridermal actin microridge system(Raman et al. 2016).

Given the fact that actin microridges are Arp2/3 dependent structures they can serve an as excellent new model system to study branched actin networks as well as the cross-talk between other pathways such as cell polarity pathways or other cytoskeletal elements and the actin cytoskeleton in vivo.

### Microvilli

Microvilli are cylindrical actin protrusions that are found in several cell types, most notably forming the brush border of intestinal enterocytes (Crawley et al. 2014a). They are thought to increase surface for absorption of nutrients, as well as serve as a reservoir of actin and membrane for wound healing (Ubelmann et al. 2013; Crawley et al. 2014a). The mouse microvillar system has served to study the functions of various actin bundling proteins in vivo especially, fimbrin/plastin, villin and espin as well as myosin 1 and ezrin in actin membrane crosslinking (Revenu et al. 2012; Tyska et al. 2005; Saotome et al. 2004). More recently, the mechanisms that generate microvilli of uniform length have been revealed and are a consequence of inter-microvillar coupling by proto-cadherins (Crawley et al. 2014b).

The zebrafish intestine is functionally homologous to that of mammals (Brugman 2016). It possesses a visually similar brush border to that of the mouse, with densely packed microvilli above a terminal web. However, till date, this structure has not been studied for its actin regulation, though using forward genetic screens some mutants such as *slimjim* were identified that show a reduced number of microvilli (Pack et al. 1996). The zebrafish *goosepimples* (Myosin Vb) mutant has reduced microvillar length, and microvilli inside internal inclusions (Sidhaye et al. 2016). This was shown using dissection and staining of the larval intestine post fixation, cryo-sectioning, and confocal imaging, as well as standard electron microscopy. This is reminiscent of human microvillus inclusion disease (MVID) and is similar to the various MVID mouse models (Schneeberger et al. 2015; Carton-Garcia et al. 2015; Weis et al. 2016; Ameen and Salas 2000). This further reflects the homology between the mammalian and the zebrafish enterocyte and brush border systems. Such zebrafish models not only have the ability to delineate actin biology but also the pathophysiology of disease. Live imaging in the mammalian intestine is difficult, on the other hand, especially in combination with light sheet microscopy and the transparent nature of albino mutant transgenic models, mechanisms by which changes to the actin cytoskeleton occur will become obvious.

Apart from the intestine, the pronephros is one of the other sites where microvilli are found. Here, it has been shown that the actin-binding peptide Peptidylglycine α-amidating monooxygenase (PAM), is necessary for the formation of normal microvilli (Kumar et al. 2018). Similarly, while modified microvilli called ‘stereocilia’ are found in the inner ear in mouse models, rendering imaging difficult in these systems, in zebrafish, stereocilia are found on the outer surface, as part of the lateral line sense organ as well as the ear or otic vesicle. These structures can be imaged relatively easily and can serve as a suitable model for studying actin biology (Hwang et al. 2015; Chitnis et al. 2012).

### Early development

A process that requires actin during the earliest stages post-fertilization is the segregation of ooplasm from the yolk to the nascent animal pole by means of streaming. It has been recently shown that the fertilized zebrafish oocyte experiences waves of actin polymerisation from the animal to the vegetal pole, and this gradient results in actin flows towards the animal pole that drive the streaming of ooplasm towards this end. The yolk granules are kept at the vegetal pole by means of another actin-based mechanism which is the formation of actin comet-like structures (Shamipour et al. 2019). Actin dynamics has been studied in oocytes and early embryos using a Moesin-GFP fusion (Nukada et al. 2015). Germ plasm segregation and localisation of many maternal RNAs in the early embryo have been found to be actin-dependent (Eno and Pelegri 2018; Theusch et al. 2006; Eno et al. 2019; Nair et al. 2013). Imaging actin dynamics in real time during germ plasm segregation and maternal RNA localisation can provide new insights.

Another prominent actin system during development is the actin ring found during epiboly. A circumferential acto-myosin ring forms near the margin of the yolk and enveloping layer, and is present within the yolk cell (Schwayer et al. 2016). Cytochalasin B-treated embryos showed a failure of epiboly suggesting that actin plays a key role in driving epiboly movements (Cheng et al. 2004). The formin zDia2 and Profilin1a proteins appear to play a role in forming this ring downstream of RhoGTPase (Lai et al. 2008). The ring is under tension and apart from circumferential contraction its flow-friction motor activity can drive epiboly (Behrndt et al. 2012). These findings were obtained by live imaging of Utrophin-CH domain transgenic animals, the use of drugs such as Cytochalasins and jasplakinolide, as well as laser ablation and theory, among other more specialised techniques.

## Conclusions

Though a wide variety of tools to image and modulate actin in vivo and in fixed samples exist, many of them have not yet found widespread usage in the zebrafish model system.

Despite zebrafish being a major model organism for several decades now and despite path breaking work with regards to the cytoskeleton, physical forces and development being performed in fish, detailed analysis of the zebrafish fish actins and their biochemical properties has not yet been performed. This might be due in part to conventional difficulties in purifying actin from native sources and also the fact that zebrafish have been widely used for its strength in genetics and not biochemistry. Nonetheless with the advent of pick-ya actin, one hopes that pure zebrafish actins will soon be purified and their properties studied. In particular, how the N-terminal modifications alter biochemical properties and interactions with actin binding proteins relative to mammalian actins will enhance our understanding of the N-terminus of actins in general.

The zebrafish has significant advantages compared to other vertebrate models when it comes to imaging. This is of benefit when studying the different model systems for actin biology, such as actin microridges. These benefits should be exploited to obtain new insight into the mechanisms by which branched actin structures are formed, how the size of actin based protrusions are controlled as well as how different cytoskeletal filaments co-ordinate their activities. Additionally, the ease of chemical genetics in zebrafish makes the screening of large molecular libraries for phenotypes in actin structures a possibility (Kaufman et al. 2009). Since actin structures such as the microridge have not been fully exploited, forward genetic screens to look at phenotypes of the microridge might reveal new interactors and regulators of the actin cytoskeleton. Finally, newer tools such as single cell RNAseq and proteomics also hold great promise in enabling a more detailed understanding of the actin cytoskeleton.

In the year 2020, actin in the zebrafish has remained relatively untouched and the field is ripe with exciting opportunities and low hanging fruit. We anticipate that in the following decades the humble zebrafish will revolutionise the actin biology field.
